# Functionalization of Emulsion Interfaces: Surface Chemistry Made Liquid

**DOI:** 10.1002/chem.202403501

**Published:** 2024-11-27

**Authors:** Clémence Courrégelongue, Damien Baigl

**Affiliations:** ^1^ PASTEUR, Department of Chemistry, Ecole Normale Supérieure PSL University, Sorbonne Université, CNRS 75005 Paris France

**Keywords:** Soft interface, Functionalization, Coating, Drop, Functional emulsifier

## Abstract

Disperse systems, and emulsions in particular, are currently massively used in fields as varied as food industry, cosmetics, health care and environmentally‐friendly materials. To meet increasingly precise needs or targeted applications, these systems need to be endowed with new functionalities at their interfaces, in addition to their composition and structural properties. However, due to the fragility of drops and the low reactivity of their surface, conventional solid surface chemistry cannot be used for such a purpose. Several specific emulsion interface functionalization techniques have thus been developed for targeted systems and applications, but a general framework has yet to be drawn. In this review, we attempt to present these methods in a unified way through the prism of what we may call “liquid surface chemistry”. We propose to categorize existing methods into drop‐coating strategies, including layer‐by‐layer techniques and polymer coating, with a particular focus on polydopamine, and emulsifier‐carrier approaches involving particles and/or amphiphilic molecules. They are discussed in a transversal way, highlighting the underlying physico‐chemical principles and providing a comparative analysis of their advantages, current limitations and potential for improvement. We also propose future directions and opportunities, involving for instance DNA‐based programmability or artificial intelligence, which could make liquid surface chemistry more versatile and controlled.

## Graphical abstract

Emulsions are widely used disperse liquid/liquid systems but usually carrying inert interfaces. Here, we overview the existing methods to functionalize their interfaces, which we categorize into coating strategies and methods based on emulsifiers as function carriers. We emphasize the importance of their liquid nature, from current challenges to perspectives for future studies and applications.

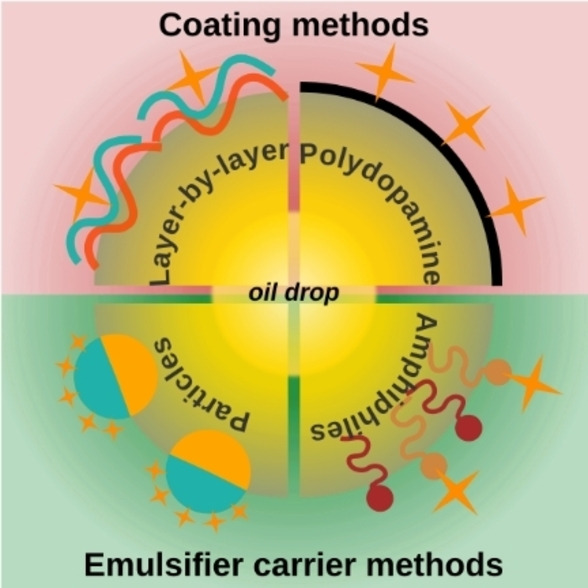

## 1 Introduction

As defined by IUPAC in 1972,^[1]^ an emulsion is the dispersion of a liquid in another liquid, both of them being non‐miscible. Naturally occurring, such as mammalian milk, emulsions are also, in many cases, human‐made, whether in everyday operations (e. g., the preparation of mayonnaise) or in large‐scale industrial processes. Natural and synthetic emulsions are used in a wide range of application domains including nutrition, cosmetics and health care. This strong societal and economic impact drives a continuous demand for more precise properties or targeted applications. It can go from the preservation of nutrients in complex food engineering^[2]^ to specific biosensors for medical purpose.^[3]^ By enabling the stable dispersion of an organic phase in an aqueous medium, emulsions also constitute a widely‐used solution for reducing the toxicity and environmental impact of modern formulations. In addition, emulsions are soft and deformable materials presenting a substantial quantity of interfaces. In the context of the current environmental crisis, they hold great promises for timely and important applications such as green interfacial catalysis using enzymes^[4]^ or water decontamination.^[5]^ To achieve these different goals, the intrinsic properties brought by the structural characteristics and chemical composition of emulsions may not be enough, requiring additional specific functionalities to be brought to their interfaces. The functionalization of emulsions interface thus appears as a critical step not only to expand the repertoire of properties emulsions can attain, but also to enable a range of applications that would otherwise not be possible.

Unlike solid substrates, for which reliable functionalization protocols have been established and optimized for a few decades, the development of robust methods for functionalizing liquid surfaces remains an ongoing and highly desired challenge. Yet a number of methods have been developed, which allow one to consider that, although in a liquid state, an emulsion interface can be functionalized in a user‐defined manner. We note, however, that this has been achieved mainly in a case‐specific context, resulting in the absence of a general framework for tuning the surface characteristics of a given emulsion into a set of desired properties. Hence, a few notable reviews devoted to the functionalization of liquid/liquid interfaces have been produced, but always focusing on a specific type of method, such as approaches based on layer‐by‐layer assembly[^6^, ^7^] or the use of Janus particles,^[8]^ or on a specific field of application, especially in the food and safety industries.[^7^, ^9^] What is lacking is an overview that would not only enable the various methods to be classified, compared and their advantages and limitations discussed, but also guide any user in the search for the most appropriate method to bring a desired function to a given emulsion of interest. This review attempts to address this need, by highlighting the concept of liquid surface chemistry. After outlining the major challenges and interests, the review presents the drop‐coating methods that are divided into layer‐by‐layer techniques and use of polymer coating, especially with polydopamine. Approaches based on emulsifier carriers are then discussed, from the use of particles to the exploitation of amphiphilic molecules. The following section is dedicated to a transversal discussion through a comparative analysis based on physico‐chemical considerations. The conclusion is then devoted to a brief summary and discussion of future prospects for emulsion functionalization.

## 2 Context and Challenges

### 2.1 Enhanced Properties Through Interface Functionalization

Following a conventional classification,^[7]^ emulsion properties can be categorized according to three main characteristics:

- Drop characteristics: composition, size distribution, or physical state of the droplets.
- Phase characteristics: Density, viscosity, controlled by the nature of the liquid and/or by addition of additives to the phases.
- Interfacial characteristics: interfacial tension, thickness, electrical charge, polarity, responsiveness or specific properties of the interfacial layer.

Each of these characteristics or their combination generally determines the main features of the emulsion, including for instance its chemical, rheological, electrical, magnetic and/or optical properties. Drop and phase characteristics can be regarded as intrinsic properties that are either implemented during emulsion preparation, or addressed by an external action, e. g., shear or heat treatment, on already formed emulsions. Decades of scientific research and industrial development have led to robust formulation and handling principles enabling control of these properties, which will not be discussed in this review. Here, we consider more specifically the situations where emulsions already have a well‐defined set of intrinsic properties (droplet and phase characteristics), but where additional features, such fluorescence, magnetism, detection or catalysis, are sought to enhance their naked properties. This can be achieved by functionalizing the liquid/liquid emulsion interface, which would otherwise remain a passive entity, and constitutes the main scope of this review.

### 2.2 Functionalization Beyond Stimulus‐Responsive Systems

A stimulus‐responsive emulsion is a system in which the emulsifier, usually a surfactant or a particle, reacts to an external signal, resulting in a change in the emulsion properties and usually its destabilization. There are numerous reviews about surfactants[^10^, ^11^] and particles[^12^, ^13^] that can respond to *stimuli* such as light,[^10^, ^11^, ^13^, ^14^, ^15^, ^16^, ^17^, ^18^, ^19^] temperature,[^12^, ^13^] a change in redox potential[^10^, ^11^] or the pH[^11^, ^12^, ^20^] of the medium. For surfactants, the *stimulus* generally leads to a change in the molecule configuration, which in turn affects its interfacial activity and thus its ability to stabilize the emulsion. In the case of particles, the stimulus generally induces a change in the wettability and anchoring of the particle at the interface, or leads to particle degradation. In both surfactants and particles cases, these changes can lead to partial or complete coalescence phenomena, resulting in a shift in drop distribution towards larger sizes, or even total destabilization of the emulsion. These systems are interesting because they can adapt to external stimuli or environmental modifications in an autonomous or directed manner, making them excellent candidates for encapsulation and targeted release applications. As these methods are already well documented, in this review we specifically focus on the situation of well‐defined emulsions with stable interfaces, for which properties beyond stimulus reactivity are sought to be added, such as fluorescence, recognition, catalysis or detection.

### 2.3 Challenges Dealing with Emulsion Nature

Emulsions are metastable systems as the interfacial energy of emulsion drops is reduced by their coalescence, because this reduces the system interfacial area. This phenomenon can be slowed down by decreasing the interfacial energy (e. g., by adding surfactants), by reducing the interfacial area between liquids (e. g., by anchoring particles), or by a combination of both effects (a typical situation with proteins). This stabilization is only kinetic, which means that drops remain highly sensitive and prone to destabilization, and must be handled with great care to maintain their integrity. This strongly contrasts with solid substrates that can be easily manipulated even under harsh functionalization (e. g. surface activation, silanization) and purification (e. g., centrifugation) conditions.

In addition, a solid substrate exhibits enormous surface reactivity due to the loss of cohesion for the upper plane of atoms. The same phenomenon occurs with a liquid interface, but the cohesion of a solid is greater than that of a fluid, resulting in higher surface energy and hence greater reactivity for solids. This high reactivity is usually recovered by a first surface activation step, which consists of removing all adsorbed species and oxides that have formed on the surface to facilitate subsequent functionalization.

Moreover, molecule motility and diffusivity are greater at a liquid interface than on a solid substrate. This can complicate the grafting of molecules if the initiation sites are not well positioned on the surface. Finally, solid surface chemistry can exploit some specific interactions, such as the use of thiol chemistry on gold surfaces, or silanization on substrates exposing hydroxyl groups. These reactions are not directly accessible at an oil‐water interface.

For all these reasons, conventional solid surface chemistry protocols cannot be applied *per se* on liquid surfaces, and new strategies must be adapted or devised to undertake the challenge of liquid/liquid interface functionalization.

## 3 Coating Methods

One approach to the functionalization of droplets involves treating the oil/water interface as a surface that can be coated. Because they are fluid and composed of liquids that are usually chemically inert (e. g., oil), emulsion liquid/liquid interfaces are less stable and reactive than solid substrates, which can be easily functionalized using surface chemistry techniques (e. g., silanization, self‐assembled monolayers) or coating processes, such as drop casting, dip‐coating, capillary assembly or spin‐coating. Hence, methods have been specifically adapted for the coating of soft systems.

### 3.1 Layer‐By‐Layer Technique

A first method is the layer‐by‐layer (LbL) technique, which consists in physically adsorbing successive layers onto a stable emulsion to achieve its post‐functionalization.^[6]^ The emulsion, usually a direct oil‐in‐water emulsion, must enable this physical adsorption on the droplet surface, either by promoting intermolecular interactions (e. g., hydrogen bonds, van der Walls forces), or by presenting a residual surface charge. This approach enables the formation of successive layers around the droplets upon addition of polyelectrolytes, particles, proteins or polymers.

**Figure 1:**
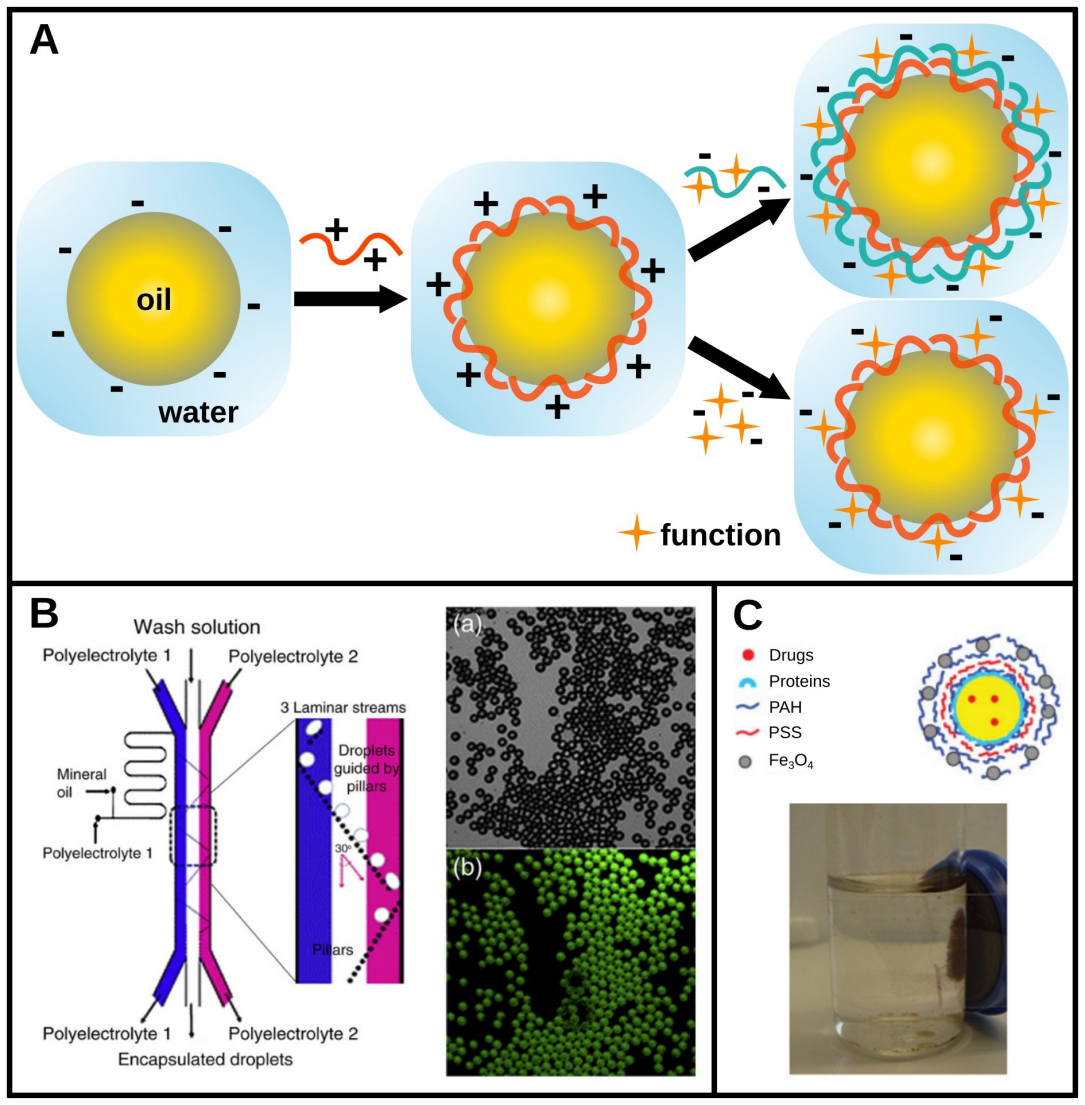
Layer‐by‐layer (LbL) assembly on drops: functions brought by a multilayer coating process. A. Schematic diagram presenting the layer‐by‐layer method in the case of an electrostatic adsorption. A polyelectrolyte adsorbs on an emulsion of opposite surface charge. Non‐flat adsorption leads to an overcharging of the surface that enables the adsorption of another oppositely charged compound (polyelectrolyte, particles, etc.). This new compound can be functional itself or be the support of an embedded function. B. The process can be generated by microfluidics to control the absorbed layers thanks to a fixed residence time and a rinsing step in between two layers. The polyelectrolyte adsorption can be monitored by fluorescence microscopy, which confirms the presence of a new fluorescence functionality at the drop surfaces. Adapted with permission of RSC, from [44]; permission conveyed through Copyright Clearance Center, Inc. C. *Top*: Schematic illustration of an emulsion functionalized with magnetic particles through electrostatic adsorption *via* a LbL approach. *Bottom*: the resulting drops are attracted to a magnet demonstrating their successful functionalization. Adapted with permission of Wiley, from [22]; permission conveyed through Copyright Clearance Center, Inc.

Most examples in the literature involve a first step in which drops carrying a non‐zero surface charge are brought into contact with polyelectrolytes or particles of opposite charge. Bringing a polyelectrolyte (PE) into contact with an oppositely charged emulsion drop results in the adsorption of a PE monolayer (Figure 1A). Conformational entropy usually leads to non‐flat adsorption and overcharging of the surface by the PE. In that case, after rinsing off the excess of the first PE, a second layer of oppositely charged particles or PE can be added. This process can be repeated as long as the coated surface remains sufficiently charged, allowing layers to be added sequentially around the drop and forming a multilayer coating. This coating can serve as a simple protective shell, or impart new functionalities to the emulsion if one or more of the adsorbed layers are functional.^[6]^

This method can be used regardless of the type of emulsifier stabilizing the emulsion, whether surfactants,^[21]^ proteins^[22]^ or particles,^[23]^ as long as the droplet interface is capable of physical interactions due to its charge or surface molecular groups.

The multilayer coating is typically realized by successively adding the species to be adsorbed to the continuous phase, with rinsing steps in between, which involves many drop manipulations and potential emulsion destabilization. This process can be facilitated by performing the successive coating/rising steps in a single microfluidic device,^[44]^ as exemplified in Figure 1B. By exploiting the laminar flow conditions at a high Péclet number, drops can be successively guided through different unmixed streams (e. g., PE1, rinsing solution, PE2) with a defined residence time in each solution, thus reproducibly forming a controlled multilayer coating on each drop.

This type of coating is now well established and used to create shells of controlled thickness around the drops, thereby increasing the stability of the emulsions against coalescence, as shown in the review of Shchukina and Shchukin.^[6]^ The coatings added can be specific, for instance to protect drops from environmental stress such as low temperatures in freeze/thaw processes,[^26^, ^27^] or variations in pH,^[24]^ ionic strength^[24]^ or oxidation.^[25]^ These types of protection are of particular interest in the food industry, where emulsions have to withstand a variety of processing and manufacturing conditions that can be harsh and detrimental to emulsion integrity. In this case, coating is usually achieved through interactions between proteins and polysaccharides which firstly stabilize the drops and then protect them from potential aggression by their environment.[^7^, ^9^] The main objective of this type of coating is therefore not to add new functionality to the drops, but above all to increase their stability.

In addition to this important field of application, it is also possible to exploit the LbL coating of drop surfaces to bring new functions to emulsions. For instance, it has been demonstrated that a layer of metal particles could be inserted between two layers of polyelectrolytes adsorbed on drop surfaces, thus imparting new magnetic properties to the emulsion^[22]^ (Figure 1C). Following this way, the drops could be manipulated using a magnet without modifying the drop interior, making the principle not only minimally invasive but also potentially applicable to many kinds of drop composition.

### 3.2 Polymer Coating

With the previous strategy, pre‐existing polymers are adsorbed on the drop surface to modify its functions. An alternative consists in directly polymerizing a monomer on the drop surface to create a coating that can serve either as is or as a basis for further functionalization. Interfacial polymerization is a commonly used strategy to create polymeric shells with tailored properties.^[45]^ This usually requires that at least one of the reagents is part of the emulsion system and leads to novel entities of interest, such as capsules, which will not be discussed here. In this review, we focus instead on coating methods that could be applied to different emulsion formulations and where drops remain the entities of interest after their functionalization.

#### 3.2.1 An Unspecific Adhesion Platform: Polydopamine

To avoid adapting the polymerization strategy for each emulsion of interest, it is valuable to look for an unspecific coating method. In case of solid substrate, a widely used strategy is the polydopamine coating, which distinguishes by its capability to coat a broad variety of surfaces.[^46^, ^47^, ^48^] This polydopamine layer possesses a number of intrinsic physicochemical properties, including optical properties (absorption and fluorescence), electrical conductivity, surface wetting modification and redox capability (metal ion reducer),[^46^, ^47^] which directly bring functionalities to the coating. It can also serve as a starting point for the chemical or physical anchoring of new molecules. Although it has mainly been applied to solid substrates, some teams have shown that it can be extended to soft interfaces by polymerizing dopamine directly at the surface of drops.

#### 3.2.2 Drop Coating with Polydopamine

**Figure 2:**
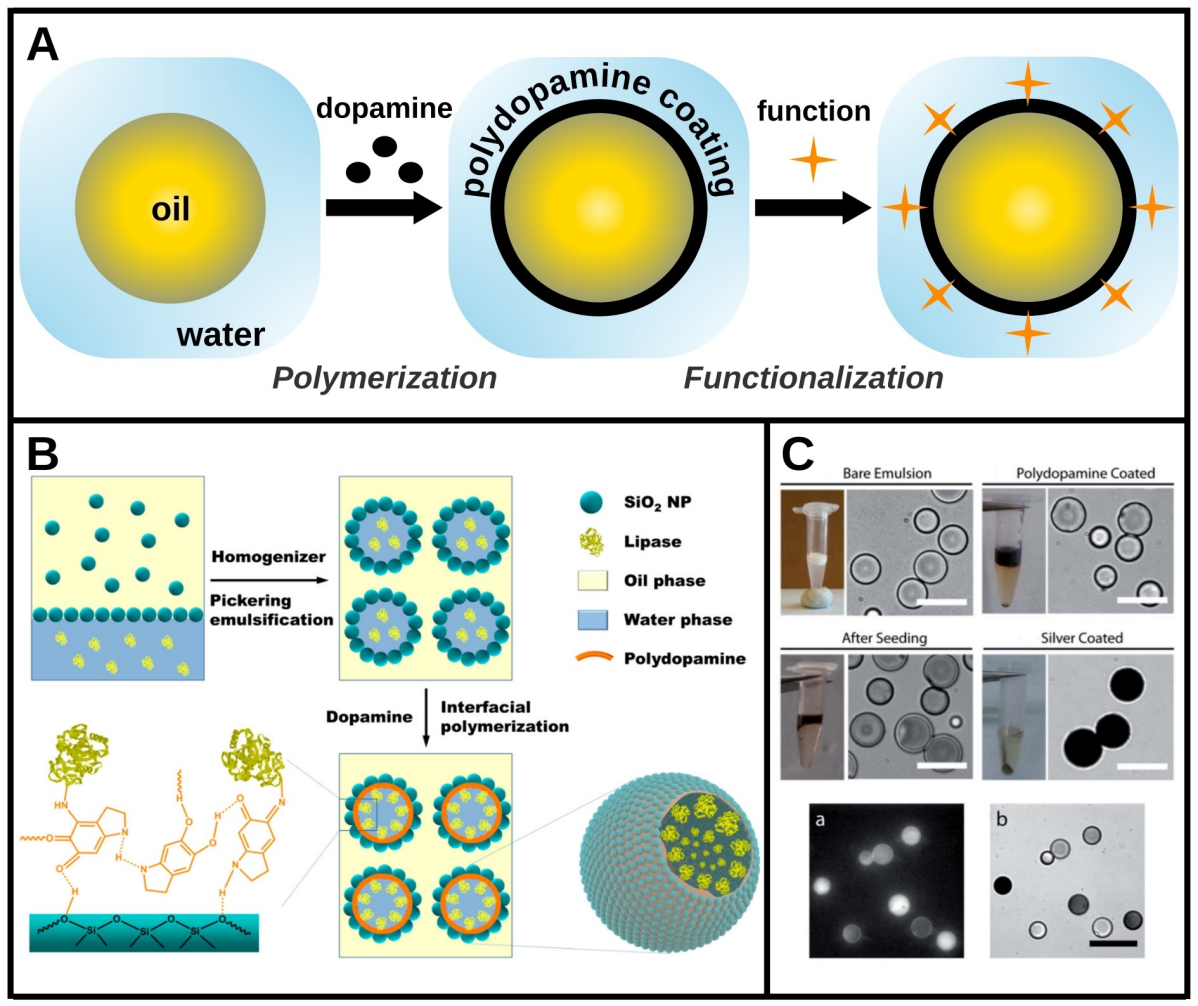
Polydopamine coating of emulsions: an interfacial polymerization bringing new functions. A. Schematic illustration of an emulsion functionalization through polydopamine coating. The first step consists in the polymerization of dopamine at the oil‐water interface to form a polydopamine layer in proper conditions (basic pH and oxidizing environment). This polydopamine layer not only brings its own set of properties but can also be used for the adsorption or covalent grafting of new function to the interface. B. Schematic diagram of the formation of a Pickering water/oil emulsion followed by its functionalization through polydopamine coating. This leads to the covalent linking of an enzyme at the interface that brings a catalytic function to the emulsion. Reprinted with permission from [4]. Copyright 2015 American Chemical Society. C. *Top*: Presentation of the successive steps to achieve the silver coating of oil drops through an interfacial polydopamine layer formation and the electrostatic adsorption of silver on it. *Bottom*: Acquisition of a new fluorescence activity as shown by fluorescence (left) and transmission (right) microscopy. Adapted with permission of RSC, from [28]; permission conveyed through Copyright Clearance Center, Inc.

Drop coating with polydopamine is carried out on preformed stable droplets.[^4^, ^28^, ^29^, ^49^, ^50^] It consists in introducing dopamine into the continuous phase under conditions favorable to its polymerization: a slightly basic pH and an oxidizing environment (directly via the oxygen atoms present in the liquid phases[^4^, ^29^, ^49^, ^50^] or with an oxidizing agent^[28]^). Once this polymerization step is complete, a functionalization step can be performed by chemical or physical binding of species to the polydopamine layer (Figure 2A). During polymerization, dopamine oxidizes and certain catechol groups form quinones. Both types of groups are therefore present on the surface of the coating and can form H‐bonds with proton donors or acceptors. In addition, quinones can react chemically with amines (Michael addition or Schiff base formation) or thiols (Michael addition), enabling functional molecules to be covalently linked to the polydopamine layer.^[46]^

This method can be applied to drops of various sizes stabilized by different types of emulsifiers (surfactants, particles, polymers).[^4^, ^28^, ^29^, ^49^, ^50^] It is generally applied to direct oil‐in‐water emulsions, as shown in Figure 2A, with dopamine in the continuous aqueous phase, but can also be applied to indirect emulsions,^[4]^ as highlighted in Figure 2B. In the latter case, the dopamine was added in powder form and migrated to the aqueous phase where it polymerized. In addition, the emulsion was stabilized by silica particles, for which dopamine has high affinity. This allowed the particle layer at the interface to act as a support for the polydopamine layer, ensuring that it was uniformly deposited across the interface as it polymerized in the aqueous phase. During polymerization, quinone groups were formed and lipases dispersed in the aqueous phase prior to emulsification were immobilized on the layer by chemical bonding via their amines (Figure 2B). Interestingly, it was found that the lipase enzymatic activity was greater when immobilized at the drop interface than at the same concentration in solution, not only demonstrating the possibility to bring catalytic properties to initially inert liquid interfaces, but also highlighting the interest in using liquid/liquid interface functionalization for improved protein manipulation and/or enhanced catalysis.

Another example of polydopamine functionalization is presented in Figure 2C, where soybean oil drops stabilized by a copolymer and an ionic surfactant were coated with a polydopamine layer.^[28]^ This layer had a negative surface charge onto which silver ions added to the aqueous phase accumulated. A reducing agent was then added to form a silver layer on top of the polydopamine layer. In addition, this silver coating exhibited strong fluorescence emission with reduced photobleaching. In this case, the polydopamine coating proved to be a smart solution for both metallizing liquid interfaces and bringing stable fluorescence to drops that were initially optically inactive.

In summary, both the layer‐by‐layer technique and dopamine polymerization constitute efficient and versatile solutions for coating liquid interfaces. Inspired by protocols usually operated on solid surfaces, they have proved adaptable to the functionalization of liquid/liquid interfaces, provided that emulsions are stable enough to retain their integrity under coating conditions. Coating layers can directly provide a set of desired functions and/or serve as reactive substrates for the physical or chemical grafting of new functional entity, ensuring versatility and a good potential for multi‐functionality.

## 4 Emulsifiers as Functionality Carriers

In the vast majority of cases, the formulation of an emulsion requires the use of one or more stabilizing agents. Hence, another possibility for functionalizing liquid interfaces consists in harnessing emulsifiers as carrier species for the desired functionality. Prior to emulsification, the stabilizing agent is modified, either to carry a functional entity directly, or to expose a reactive group that will be exploited after emulsification to bring the new function to the interface.

### 4.1 Particles

It is now well established that amphiphilic particles constitute a valuable type of emulsifier thanks to their virtually irreversible anchoring at oil/water interfaces. This anchoring leads to the well‐defined Ramsden or Pickering emulsions,[^51^, ^52^] composed of highly stable drops of controlled sizes thanks to the phenomenon of limited coalescence, which is of particular interest for material design.^[53]^ Interestingly, with advances in surface chemistry and particle manufacturing, new functions can be incorporated into particle by grafting on their surface or by modifying their intrinsic composition. After the emulsification step, this function is directly appended to the particle‐laden interface.

**Figure 3:**
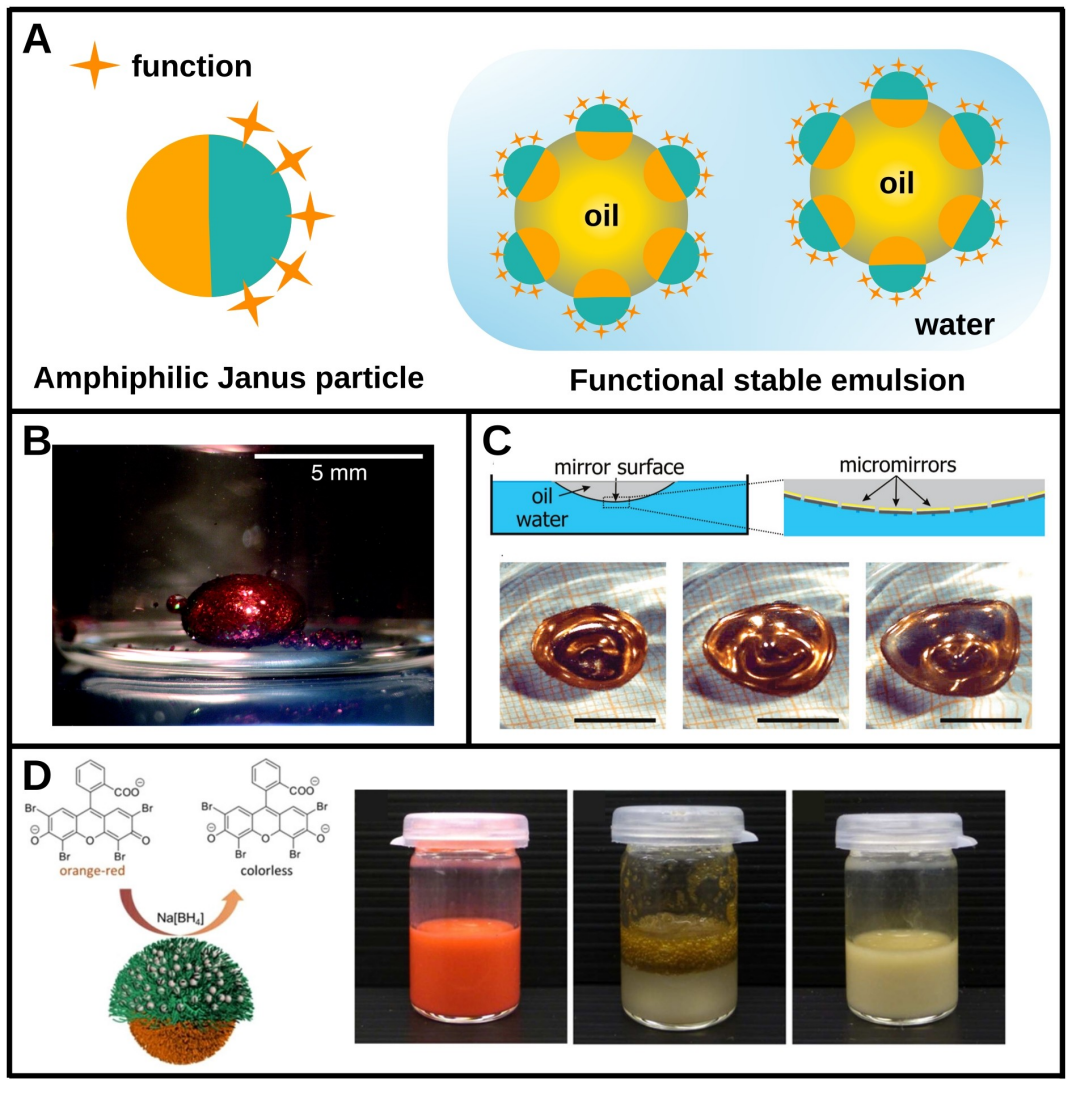
Janus particles: a strong anchoring at liquid/liquid interfaces to append new functionalities. A. Schematic illustration of an emulsion functionalization with Janus decorated particles. The particles are designed prior emulsification to adjust their amphiphilicity and attach new functions at the desired position. Then they will stabilize the emulsion and anchor the new functionality at the interface. B. Photography of a bi‐color mirror formed with Janus tiles at an oil/water interface with the red hydrophilic part towards the exterior. Reprinted with permission from [35] Copyright (2003) National Academy of Sciences, U.S.A. C. (Top) Schematic illustration of a mirror formed at an oil/water interface by the anchoring of Janus tiles nanoparticles. (Bottom) Images of the liquid mirror under acoustic excitation (450 ms between frames) where it stays intact and reflective. Scale bar: 1 cm. Adapted with permission from [37]. Copyright 2009 American Chemical Society. D. (Left) Schematic diagram of a reduction reaction catalysed by the Janus particle. (Right) Photographs of the reduction reaction in a water/oil emulsion stabilized by the particles. Adapted with permission from [33]. Copyright 2015 American Chemical Society.

Efficient particle anchoring at an oil/water interface requires proper adjustment of the particle surface hydrophobic/hydrophilic balance. This amphiphilicity can be provided by hydrophobic or hydrophilic moieties that are either uniformly distributed over the particle surface, or partitioned into two distinct hemispheres to form the so‐called Janus particles. Whether spherical or not, symmetric or not, Janus particles are characterized by a stark difference in composition or coating between the two parts, which allows differentiated wetting and thus strong anchoring at a hydrophilic/hydrophobic interface, making them excellent stabilizers of liquid/liquid, and especially water/oil, interfaces.[^54^, ^55^] In addition, it is possible to selectively coat one of the faces of these particles^[56]^ while maintaining amphiphilicity and anchoring at the interface, thus providing new functions with a specific exposure to one of the liquids, as shown in Figure 3A. Particles are often used to structure and functionalize solid substrates[^57^, ^58^] for optical, electronic, detection or biomedical applications. The strong anchoring of Janus particles at soft interfaces allows to transpose those techniques to functionalize liquid/liquid interfaces.^[8]^

This ability to attach a function at a drop interface by grafting it to irreversibly anchored particles, has made it possible, for instance, to form optically active drops.[^35^, ^36^, ^37^] For example, porous particles consisting of a two‐color double mirror with a red hydrophilic face and a green hydrophobic face were formed.^[35]^ These particles were adsorbed at a liquid interface between heptane and water, forming red drops on the outside (facing water) with a green mirror on the inside (heptane phase), as shown in Figure 3B. The selectivity of the particle faces towards a hydrophobic or hydrophilic phase was monitored by changes in the optical spectra of the mirrors, making it possible to detect the hydrophobic or hydrophilic nature of a liquid. It was also possible to position micromirrors at a oil/water interface to form liquid mirrors^[37]^ as shown in Figure 3C. These mirrors are mechanically stable and resist high vibratory excitation and could therefore be used in advanced optofluidic systems such as 3D projectors.

Janus magnetic particles were also synthesized and, after emulsification and anchoring, imparted magnetic properties to the drop interface.^[38]^ The great stability of Ramsden/Pickering emulsions stabilized by Janus particles has enabled the recovery of oil drops using a magnet. Such a means of extracting oil from and oil/water medium has good potential for applications in pollution control or in the food industry.

Finally, large‐surface‐area systems, such as porous materials, foams or emulsions, are attractive entities for catalysis applications where optimization of surface exchange is sought after. Furthermore, an emulsion enables two species solubilized in two different phases to come into contact and react at the interface. In this case, the presence of a catalyst at the interface is of great interest to promote the reaction. This can be achieved by grafting a catalyst onto the particles, not necessarily of the Janus type, prior to their anchoring at the interface during emulsification, leading *in fine* to acceleration of the reaction,[^30^, ^31^, ^32^, ^33^] as illustrated in Figure 3D. The coupling of magnetic properties to this catalyst grafting allows the catalyst to be recycled and reused.^[34]^

### 4.2 Amphiphilic Molecules

Although particles are becoming increasingly popular for stabilizing emulsions, amphiphilic molecules such as surfactants and block copolymers are still widely used and studied. These emulsifiers can also be used to functionalize emulsions. Two types of use can be distinguished: direct use of the amphiphilic molecule as a function carrier, or indirect use, with the presence on the molecule of a reactive site which serves to carry out a chemical reaction or conjugation reaction after emulsification. In the following, if the molecule can be used directly for a specific function, it will be referred to as a function carrier. On the other hand, if the molecule must first react with another molecule, it will be referred to as a reactive site carrier.

#### 4.2.1 Function‐Carrier Molecules

**Figure 4:**
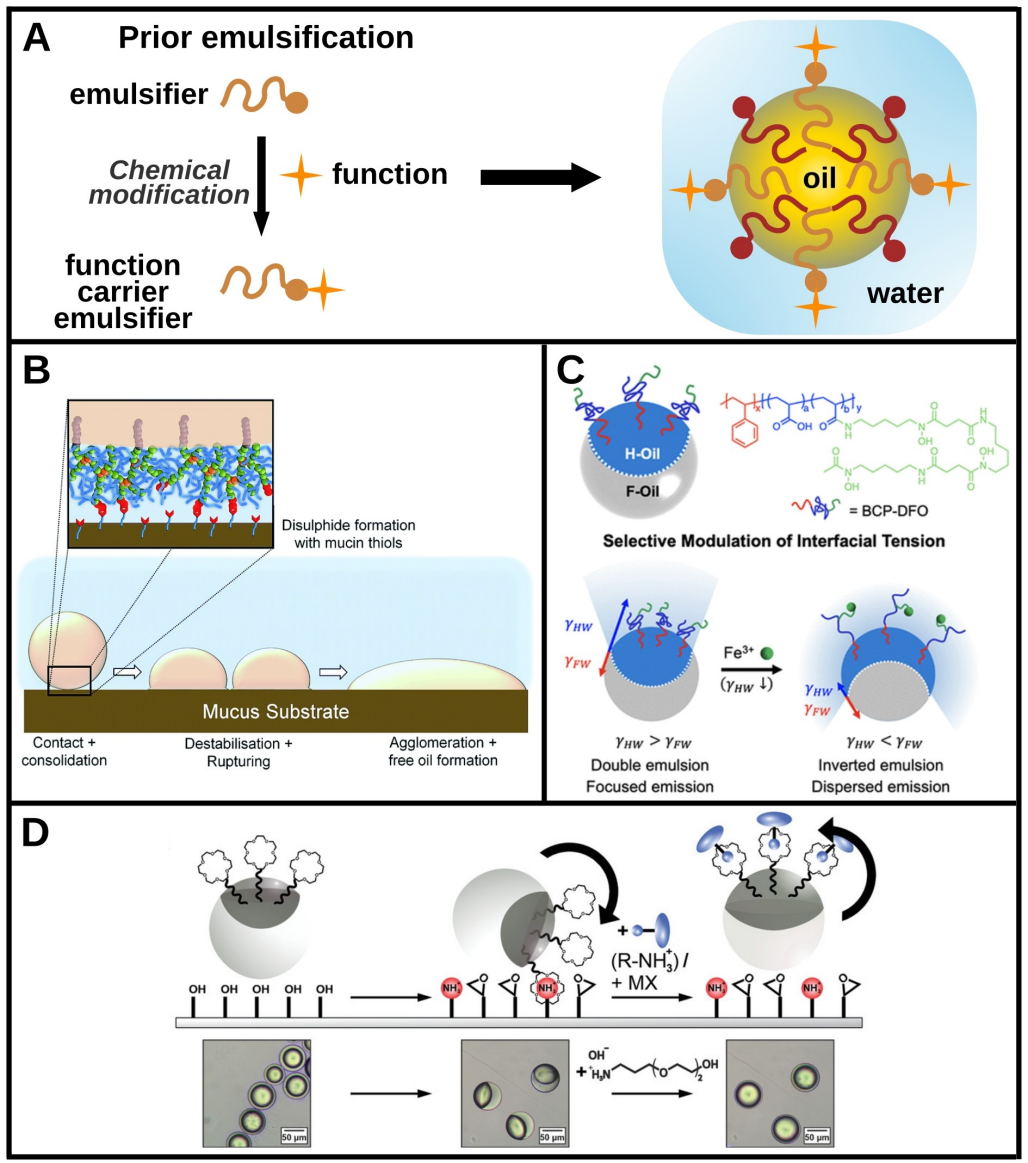
Function‐carrier emulsifiers: specific design of amphiphilic molecules to provide new functions at liquid interfaces. A. Schematic representation of a liquid interface functionalization using an amphiphilic function carrier emulsifier. An amphiphilic molecule (for example a surfactant or a block copolymer) is first modified before emulsification to carry a new function. Upon emulsification, this modified emulsifier localizes at the interface where it brings the function. B. Drops are stabilized with branched copolymers presenting thiol groups at their extremities. These thiol groups can selectively form disulfide bonds with a mucus substrate, leading to drop destabilization giving a targeting functionality to the interface. Adapted from [59] under the terms of the CC‐BY‐3.0 licence. C. Schematic illustration of a biphasic emulsion composed of a hydrocarbon (H‐oil with a fluorescent dye) and a fluorocarbon (F‐oil) in water. The emulsion is stabilized, among other surfactant molecules, by a copolymer presenting Fe^3+^ chelating sites at one ending. The chelation modifies the polymer surface activity which impacts the morphology of the emulsion and thus the fluorescent signal. This gives detection properties to the drop interface with potential applications in metal ions sensing in polluted water. Adapted with permission from [5]. Copyright 2023 American Chemical Society. D. Schematic representation and microscope images of a bi‐phasic emulsion (hydrocarbon and fluorocarbon in water) partially stabilized by crown ether‐functionalized surfactants. The drops are put into contact of a functionalized glass surface where the ether can form supramolecular complexation with the amines. This leads to a tilting of the drops while the addition of a competitive hydrophilic ammonium species re‐orientates the drops. It gives different optical properties to the system linked with those morphological changes and thus an optical detection functionality. Adapted from [39] under the terms of the CC‐BY‐4.0 licence.

During the tailoring of a stabilizing polymer or surfactant, it is possible to add groups that can provide a new function. During the emulsification step, the emulsifier accumulates at the interface where it brings the new function, as shown in Figure 4A. The diagram shows the example of an oil‐in‐water emulsion functionalized on the outer surface of the droplets. This corresponds to the majority of cases presented in the literature, for reasons mainly linked to emulsion formulation and stabilizer architecture. Indeed, direct emulsions are the most common because of the quantities required (generally a small volume of organic liquid is dispersed in a continuous aqueous phase) and the applications targeted. In addition, an amphiphilic stabilizing molecule (surfactant, polymer, lipid, etc.) is generally made up of a hydrocarbon chain facing the oil and a polar head of greater chemical versatility facing the water.

The use of block copolymers enables the controlled addition of specific chemical groups during the polymerization steps. The presence of these chemical groups can in turn bring new functionalities to the emulsions. For example, these groups can selectively bind to a specific environment for targeting applications,^[59]^ as shown in Figure 4B. In this example, thiol moieties on the branched chains of the block copolymer selectively bound to mucus, enabling specific areas to be targeted for medical applications. Another example is shown in Figure 4C, where metal chelators have been added to the chains.^[5]^ These sites were used to chelate Fe^3+^ ions for specific detection of metal ions in polluted water. A bi‐phasic emulsion, with a dispersed phase composed of immiscible fluorocarbon and hydrocarbon oils, was stabilized with those polymers supplemented by other surfactants. Interfacial tensions between the organic phases and the aqueous phase dictated the morphology of the emulsion. A fluorescent species was solubilized in only one of the two organic phases. The chelation of Fe^3+^ ions led to a change in the polymer interfacial properties and hence interfacial tensions, leading in turn to a change in emulsion morphology and fluorescence signal, allowing to optically detect the presence of Fe^3+^ ions in water. This clearly shows that the addition of a functional group to an emulsifier, making it a function carrier, can transform a passive disperse system into a functional emulsion, such as, in this case, a liquid/liquid optical ion sensor.

This functionalization principle can also be applied to surfactants in which some functional groups can be added to the polar head. Figure 4D shows the example of surfactants with a crown‐ether head which can perform host‐guest interaction with ammonium moieties or inorganic metal salts, leading to different interfacial properties for the complex.^[39]^ This can bring new functions to the emulsion, such as catalytic properties or specific binding to some substrates. In the latter case, a bi‐phasic emulsion can show optical properties with induced morphological changes as explained above.

In addition, the crown‐ether's affinity for various compounds can also make it a reactive site to perform a conjugation reaction prior to having a definitive function, paving the way for a supramolecular functionalization platform.

#### 4.2.2 Reactive Amphiphiles

**Figure 5:**
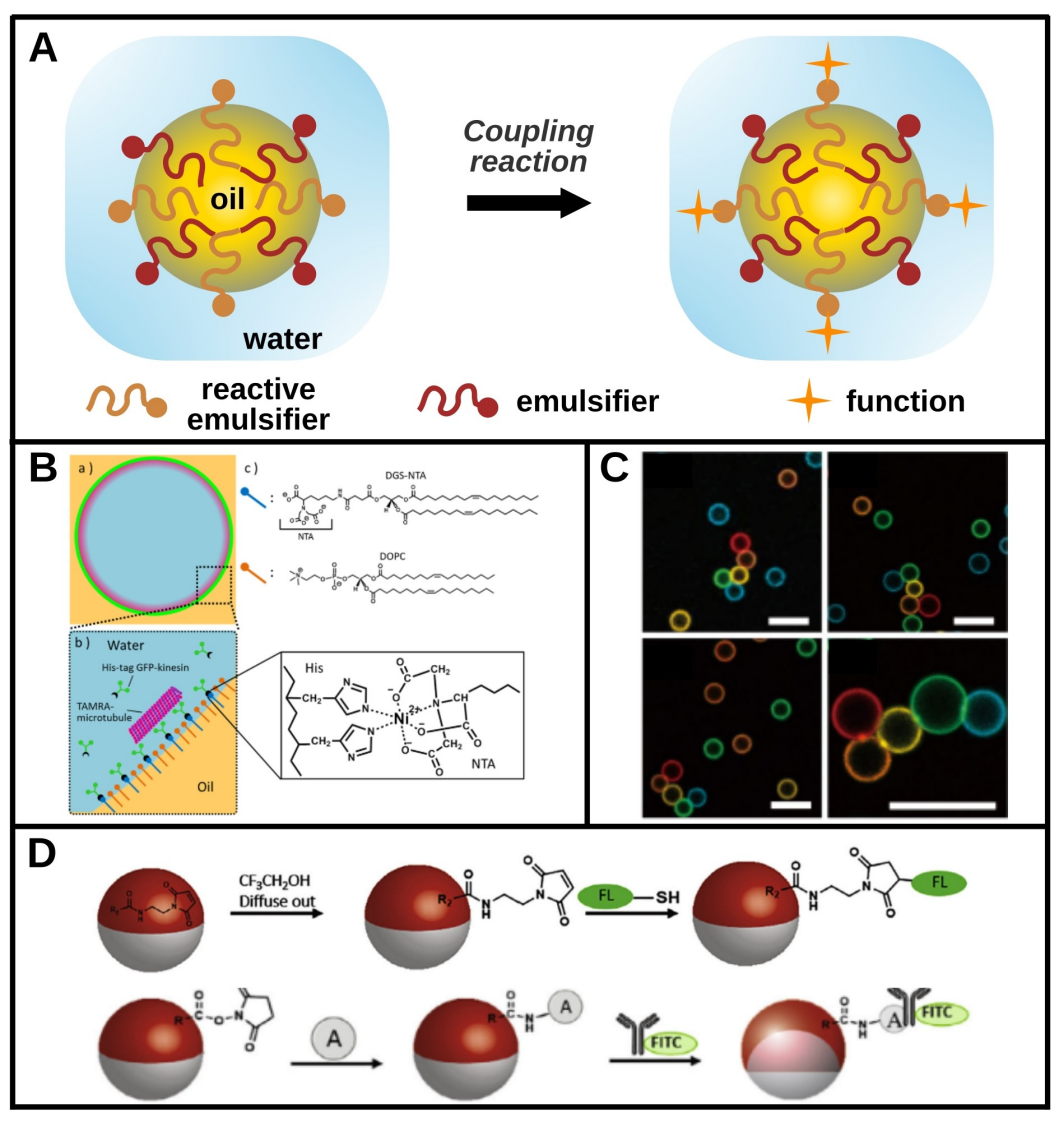
Reactive amphiphiles: post‐conjugation of emulsifiers at interfaces. A. Schematic representation of the use of a reactive site carrier molecule to functionalize an emulsion. An emulsion is formulated with amphiphilic molecules including modified reactive emulsifiers. Those molecules carry reactive sites that can be physically or chemically conjugated with new functions in a post‐functionalization process. B. Schematic illustration of the functionalization of the interior of an indirect emulsion drop by using lipids. The nitrilotriacetic acid (NTA)‐modified lipid (DGS‐NTA) is able to make a complexation reaction with histidine (HIS) residues of kinesin in the presence of Ni^2+^ ions. These kinesin proteins can interact with microtubules and enable to study their motility at a curved liquid interface. Adapted with permission from [42]. Copyright 2017 American Chemical Society. C. Confocal microscopy images of self‐assembled structures of drops coated with complementary DNA sequences. The drops are false colored with each color representing a different DNA sequence. Each sequence is designed to allow a single succession pattern for the drops. Scale bars: 10 μm. Adapted from [41] under the terms of the CC‐BY‐4.0 licence. D. Schematic of the post‐functionalization of a biphasic emulsion. An interfacial species diffuses to the interface where it can undergo a conjugation reaction to couple a fluorophore (top) and a protein A able to detect an immunoglobulin (bottom). Reprinted from [3], with permission from Elsevier.

It is also possible to consider emulsifiers as reactive intermediates for the post‐functionalization of liquid/liquid interfaces. The strategy involves formulating an emulsion with one or more molecular emulsifier(s) (surfactant, polymer, lipid, etc.), at least one of which can be described as reactive. The term “reactive” is used here to qualify a species capable of carrying out a chemical^[3]^ or coupling reaction (host‐guest interaction,^[39]^ with a metal ion,^[42]^ biotin‐streptavidin coupling,[^40^, ^41^] etc.) after being positioned at a liquid/liquid interface. This makes it possible to post‐functionalize an emulsion after its formulation, under the conditions required for post‐conjugation, as shown in the diagram in Figure 5A.

As in Figure 4A and for the same reasons, the diagram shows an oil‐in‐water emulsion functionalized in the aqueous continuous phase. The question of positioning of the reactive site on the molecule and therefore at the interface is even more critical here. Indeed, the reactive site must be accessible to form a chemical bond or coupling reaction with an ion or another molecule. For instance, the accessibility of the crown‐ether is of paramount importance to achieve post functionalization supported by supramolecular interaction, as shown in a recent report.^[39]^ The host‐guest reaction with amines enabled post conjugation with an IgG antibody and boronic acid receptors of carbohydrates, turning the emulsion drops into bioactive sensors capable of specific recognition.

The reactivity of the polar heads of stabilizers can also be used to functionalize the interior of drops in indirect emulsions,^[42]^ as shown in Figure 5B. Two types of polar‐headed lipids, DOPC and DGS‐NTA, a lipid exposing a nitrilotriacetic acid (NTA) function, were used in this case to stabilize water drops in a continuous oil phase. Ni^2+^ ions were added to the aqueous phase along with kinesin motor proteins and microtubules, resulting in the formation of a binding complex between the NTA group, Ni^2+^ ions and histidin residues of kinesin. One of the two lipids used interacted with the kinesins to form a complex around the Ni^2+^ ions. Microtubules then interacted with the kinesins and localized at the interface. This functionalization made it possible to study the mobility of microtubules on a deformable curved interface, and could facilitate the study of certain cellular phenomena at model interfaces.

It is also possible to modify a lipid upstream to fuse it with streptavidin or biotin and then exploit the strong binding between these two compounds^[60]^ to achieve post‐conjugation. This coupling between the lipid on the one hand, and a target molecule covalently linked to biotin or streptavidin on the other, anchors the target molecule to the interface and thus functionalizes it. Following this way, liquid/liquid interfaces can acquire different functionalities, such as biological recognition with antibody‐coated drops^[40]^ or self‐assembly.^[41]^ For instance, biotinylated DNA was conjugated to oil drops formulated in the presence of streptavidin‐bearing lipids to arrange DNA single strands on the drop surface. Separately prepared drops carrying different DNA sequences were then able to self‐assemble according to a programmed sequence when brought into contact, enabling DNA‐encoded, programmable and directed assembly of the functionalized drops (Figure 5C). Other methods have also been developed for drop coating with DNA. For example, the use of a reactive copolymer functionalized with azide groups, which then reacted with dibenzocyclooctyne‐functionalized DNA single strands or dyes, enabled the preparation of drops that fluoresced or were capable of selective self‐assembly.^[43]^

Finally, these emulsifiers can initiate post‐functionalization chemical reactions at liquid/liquid interfaces.[^3^, ^61^] This is achieved by adding a block copolymer or surfactant, with a hydrophobic part and a reactive hydrophilic head, to the emulsion during its preparation. Once the drops are formed, the hydrophilic head migrates to the external aqueous phase, while the hydrophobic part remains in the organic phase. Under favorable conditions, the hydrophilic head in the continuous phase can undergo chemical reactions to introduce new molecules at the interfaces. This was achieved, for instance, using thiol or azide chemistry.^[61]^ The formation of such covalent bonding after emulsification allows to bring new functions to the interface, such as fluorescence or antibody‐based biosensing, as shown in Figure 5D. In that case, the hydrophilic head of the added species consisted of either an amine or a thiol, which enabled a variety of post‐functionalization reactions, demonstrating the versatile character of the method.

## 5 Discussion

We have seen in the previous sections that the different methods for the functionalization of liquid/liquid interfaces, while sharing common features, differ in many respects, both in the way they are implemented and in the functions they can bring to the interfaces. This diversity implies, on the one hand, that a large number of liquid/liquid interfacial systems can be functionalized by proper adaptation of the most suited method and, on the other hand, that a wide spectrum of functions can be appended to liquid interfaces. To provide a transversal overview of the functionalization methods as well as to establish guidelines to identify the best functionalization strategy, we discuss in the following some key‐features that deserve consideration. The characteristics of each method in terms of these key‐features, the type of emulsifier, the nature of the added function and/or its field of applications are presented synoptically in Table 1.

**Table 1:** Overview of the different methods of emulsion interface functionalization. For each category, a list of key‐features, the nature of the emulsifier involved and the functionality/application acquired are listed.

Category		Key features	Emulsifier	Application or interface functionality
Layer‐by‐layer		Post‐functionalization method. Good versatility: access to different functions with a single emulsion but the functions need to be embedded in the layers. Need of an emulsion suitable for layer adsorption (surface charge or specific interaction) and stable under coating conditions. Multi‐step process.	Surfactants,^[21]^ proteins,^[22]^ particles^[23]^	Fluorescence, magnetism,^[22]^ resistance to specific conditions in food industry[^7^, ^9^]: pH variation,^[24]^ oxidation,^[25]^ freeze‐thaw processing,[^26^, ^27^] etc.
Polydopamine coating		Post‐functionalization method. Good versatility: access to diverse functions after emulsion coating but the desired function needs to be compatible with polydopamine chemistry. Emulsions need to be stable under polydopamine polymerization conditions (high pH and reduction conditions).	Surfactants,[^28^, ^29^] particles^[4]^	Fluorescence,^[28]^ magnetism, biosensing,^[4]^ catalysis,^[4]^ etc.
Functional particles		Access to a broad variety of functions thanks to well‐established surface chemistry and particle synthesis protocols. Necessity to change/adapt the emulsifier for each new desired function. Requires *ad hoc* particles enabling both sufficient stabilization and desired functionality. Emulsification and functionalization are done at the same time.	Amphiphilic particles[^30^, ^31^] Janus particles[^32^, ^33^, ^34^, ^35^, ^36^, ^37^, ^38^]	Catalysis,[^30^, ^31^, ^32^, ^33^, ^34^] optical properties,[^35^, ^36^, ^37^] magnetism,[^34^, ^38^] etc.
Amphiphilic molecules	Function carrier	Emulsification and functionalization are done at the same time. Requires *ad hoc* emulsifiers enabling both proper LHB maintenance and desired functionality.	Surfactants,^[39]^ lipids,[^40^, ^41^, ^42^] polymers[^5^, ^43^]	Fluorescence,^[40]^ sensing[^3^, ^5^, ^39^, ^40^] (bio‐interaction^[42]^), catalysis,^[39]^ optical properties,^[39]^ programmable assembly[^41^, ^43^]
	Reactive‐site carrier	Post‐functionalization method. Good versatility: access to different functions with a single emulsifier. Emulsions need to be stable under post‐conjugation conditions. Synthesis process can be difficult to keep the LHB while adding the reactive functionalization sites.		

### 5.1 Functionalization‐Enhanced Stability

The functionalization methods presented in this review are generally accompanied by an increase in emulsion stability, making them even more attractive. Indeed, the coating of drops is usually accompanied by a limitation of their coalescence, either by increasing the repulsion (electrostatic, steric, etc.) between them, or by increasing the mechanical stability of their interface, thus reducing the risk of fusion when two drops come into contact.[^27^, ^62^] When the function is carried out directly by an emulsifier, the addition of this species during formulation can also increase the stability of the disperse system.

### 5.2 Post‐Functionalization: A Gain in Versatility and its Limits

Some of the methods allow for post‐functionalization, which advantageously enables a desired function to be added to the target emulsion only at the time it is needed, thus avoiding the risk of function degradation, especially during emulsion pre‐storage. It also enables the formulation of a large quantity of a mother emulsion prior to its division into smaller batches that can be individually functionalized with different functions.

However, the latter option remains limited because, taken individually, each method has limited versatility, either in terms of the functions that can be added, or because of the limitations associated with their implementation. In fact, regardless of whether or not post‐functionalization is possible, each method has its own prerequisites, either in terms of preformed emulsion for coating techniques, or in terms of emulsifiers when these carry functions or conjugation intermediates. These conditions are discussed in the following section.

### 5.3 Prerequisites: Specific Constraints and Their Consequences

Layer‐by‐layer coating requires preformed drops with a residual surface charge or specific groups for physisorption.^[6]^ This limits the use of this method to specific systems, as well as its versatility, since it is impossible for an interface with given properties to be covered by any coating layer and thus access all theoretically possible functions.

The use of polydopamine is less dependent on the initial interface of the emulsion, as there is no need for specific interaction with drops.^[48]^ What limits its versatility is the functionalization of this polydopamine layer. Indeed, the functions provided must be capable of forming specific physicochemical interactions (e. g., H‐bonds or groups capable to form covalent bonds) with this polymer.[^46^, ^48^] Although post‐functionalization with different functions is possible, the range of accessible functions remains limited.

Emulsifiers need to be chemically modified to graft the desired function or to make them reactive for the desired conjugation. In the case of Janus particles, although this method enables the formation of highly stable, monodisperse emulsions with a well‐locked function at the interface, this remains the main drawback. Indeed, a new particle synthesis (or modification) is required for each function to be added.^[8]^ In addition, the need to add a function‐carrying emulsifier (particle or amphiphilic molecule) during formulation eliminates any possibility of post‐functionalization. In the case of reactive site carriers, a given type of emulsifier may enable different functionalizations to be achieved for the same starting emulsion, provided conjugation is based on the same chemical reactivity or specific affinity (e. g., biotin‐streptavidin). Nevertheless, it is necessary to change the emulsifiers as soon as one wishes to change the conjugation route.[^3^, ^41^] On the other hand, chemical modification of emulsifiers can prove tricky to introduce the reactive function or group while retaining the ability to stabilize the disperse system.

Regardless of the method, when post‐functionalization is possible, it can impose additional specific constraints. In the case of layer‐by‐layer coating, the drops have to undergo several changes of the continuous phase to ensure the proper adsorption and rinsing of the different layers.^[6]^ They must therefore be particularly stable against coalescence, so as not to lose the properties of the starting emulsion (e. g., drop size, polydispersity index). In the case of polydopamine functionalization, the drops must be able to withstand the polymerization conditions (basic and reducing environment) and then, if necessary, the conditions required for a chemical reaction on this layer to complete the functionalization.^[46]^ With reactive‐site carriers, the emulsion must also remain stable under the conditions required for the conjugation reaction. This may involve changing the continuous phase for a given buffer, rinsing steps, or the experimental conditions required for the chemical reaction between the emulsifier and the function to be grafted.[^3^, ^40^, ^41^] Therefore, these methods may allow post‐functionalization in only some specific cases.

## 6 Summary and Outlook

From fundamental soft matter research to their involvement in broad application fields ranging from biotechnology to food industry, emulsions have been actively studied and developed. This has allowed emulsion science to reach an advanced degree of maturity. Hence, robust methods are now available to properly adjust the compositions of the phases and adapt the emulsification conditions (choice of emulsifier, emulsification method), so that many physico‐chemical characteristics of emulsions (stability, rheology, properties of the disperse and continuous phases) can be fine‐tuned in a user‐defined fashion.

Emulsions are also characterized by their large number of liquid/liquid interfaces, usually between an oil phase and a water phase. These interfaces are involved in many emulsion properties, but often act primarily as inert boundaries. In order to exploit this large number of interfaces and extend the range of properties that emulsions can possess, researchers have developed methods for functionalizing these interfaces. We have shown how challenging such functionalization can be, for mainly three reasons. Firstly, liquid interfaces are often chemically unreactive. Secondly, their fluid nature makes it very difficult to permanently attach a chemical group and complicates purification steps. Finally, whatever the functionalization method, it must be compatible with maintaining emulsion integrity and stability.

We have shown that, despite these difficulties, there are a number of examples in the literature where liquid/liquid interfaces were efficiently functionalized by the successful incorporation of new functions of interest such as fluorescence, catalysis, magnetic properties, self‐assembly or recognition. This has led us to categorize these methods by distinguishing two main types of approach: one based on interface coating, the other on the use of emulsifiers as functionalization carriers. We have shown that the functionalization can directly embed the targeted property or serve as a reactive substrate for bringing the function (post‐functionalization). By taking into account the underlying physico‐chemical mechanisms specific to each method, we have highlighted the specific features of each strategy and provided a transversal discussion comparing the methods. This might contribute to a better understanding of emulsion properties as well as provide some guidelines when looking for the most suitable strategy to functionalize a given emulsion of interest.

Compared to years of development in solid surface chemistry, the functionalization of liquid interfaces remains at its infancy, with limitations as well as many interesting perspectives to be envisioned or explored. For instance, in current methods the reactivity implementation always relies on a guest (coating materials, emulsifier) localizing at the interface. It would be very interesting to explore guest‐free alternatives and invent some chemistry directly applicable at the liquid interface. We envisage that artificial intelligence approaches[^63^, ^64^, ^65^] might contribute to the identification of robust chemical reactions for such a purpose.

We also note that most of existing methods have been developed for uniform functionalization, i. e., the desired function is homogenously distributed all around the drop. To develop better mimics of living systems with precise spatio‐temporal distribution of their components and to extend the range of applications, it would be valuable to explore methods for heterogeneous functionalization of drop surfaces. One strategy could rely on using phase‐separating surfactants or lipids as functionality carriers, resulting in patchy drops with new function(s) localizing specifically on target phase(s).

Moreover, we have seen that the methods for functionalizing liquid/liquid interfaces require proper adjustment for each system of interest, making each method case‐specific and with limited versatility. It would be highly valuable to develop programmable approaches where a single protocol could lead to a variety of functions that can be selected and tuned in a user‐friendly and highly controlled fashion. Using synthetic DNA as molecular program to define structures (using DNA nanotechnology methods)[^66^, ^67^, ^68^, ^69^] or functions (via synthetic biology concepts)[^70^, ^71^, ^72^, ^73^, ^74^, ^75^] appears to us as an interesting research direction to approach such a goal,^[76]^ as a fixed chemical composition (DNA addition) could lead to a broad range of programmable functions by simply adapting the DNA sequence design.

Finally, we have overviewed here methods leading to a steady‐state functionalization: once a new function is added, it does not change nor vary any more in time. We think that looking for methods allowing reactive or dynamic functionalization of liquid interface could contribute to the development of a new generation of smart soft materials. This would include the possibility for drops to change their interfacial properties as a function of their external environment, making them capable to sense, adapt and/or optimize their behaviour in an autonomous manner. It could be also interesting to devise drops that change their surface properties as function of their content dynamics, making possible to develop new concepts for smart drop microfluidic screening and sorting.

These research directions are only some examples that would help enriching our understanding of soft matter and emulsion science as well as expand the potential of drops and emulsions in a broad range of scientific, every‐day‐life and industrial fields.

## Conflict of Interests

The authors declare no conflict of interest.

7

## Biographical Information

*Clémence Courrégelongue is a PhD candidate at Ecole Normale Supérieure (ENS) in Paris. Her research interests include emulsion science, particles and proteins at fluid interfaces and genetic encoding of soft matter properties*.

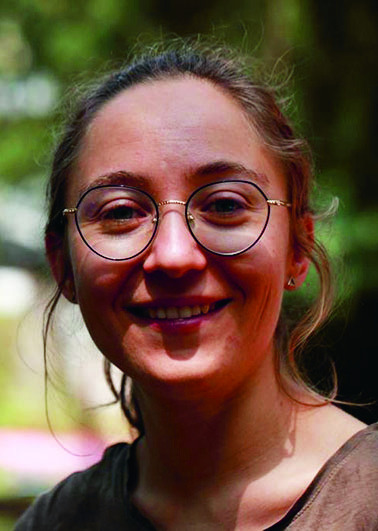

## Biographical Information

*Damien Baigl is exceptional class professor at Ecole Normale Supérieure (ENS) in Paris. After a PhD at College de France in Paris (2000–2003) and a post‐doc at Kyoto University (2003–2005), he got a permanent position at the Department of Chemistry of ENS in 2005 where he became full professor in 2010. Curiosity‐driven, he has a passion for exploration and soft matter systems. His current research interests include dynamic DNA nanotechnology, reconfigurable self‐assembly, soft synthetic biology, coffee‐ring effect for patterning and diagnostics, colloidal organization at fluid interfaces, synthetic cells, and genetic encoding of soft matter properties*.

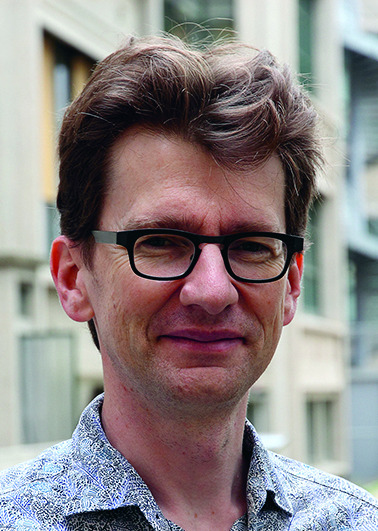

## Data Availability

Data sharing is not applicable to this article as no new data were created or analyzed in this study.
